# Prolonged Egg Supplement Advances Growing Child’s Growth and Gut Microbiota

**DOI:** 10.3390/nu15051143

**Published:** 2023-02-24

**Authors:** Sophida Suta, Apinya Surawit, Pichanun Mongkolsucharitkul, Bonggochpass Pinsawas, Thamonwan Manosan, Suphawan Ophakas, Tanyaporn Pongkunakorn, Sureeporn Pumeiam, Kitti Sranacharoenpong, Sawannee Sutheeworapong, Patcha Poungsombat, Sakda Khoomrung, Pravit Akarasereenont, Iyarit Thaipisuttikul, Bhoom Suktitipat, Korapat Mayurasakorn

**Affiliations:** 1 Population Health and Nutrition Research Group, Faculty of Medicine Siriraj Hospital, Mahidol University, Bangkok 10700, Thailand; 2 Institute of Nutrition, Mahidol University, Nakhon Pathom 73170, Thailand; 3 Systems Biology and Bioinformatics Research Unit, Pilot Plant Development and Training Institute, King Mongkut’s University of Technology Thonburi, Bangkok 10140, Thailand; 4 Metabolomics and Systems Biology, Department of Biochemistry, Faculty of Medicine Siriraj Hospital, Mahidol University, Bangkok 10700, Thailand; 5 Siriraj Metabolomics and Phenomics Center, Faculty of Medicine Siriraj Hospital, Mahidol University, Bangkok 10700, Thailand; 6 Department of Pharmacology, Faculty of Medicine Siriraj Hospital, Mahidol University, Bangkok 10700, Thailand; 7 Department of Microbiology, Faculty of Medicine Siriraj Hospital, Mahidol University, Bangkok 10700, Thailand; 8 Department of Biochemistry, Faculty of Medicine Siriraj Hospital, Mahidol University, Bangkok 10700, Thailand

**Keywords:** malnutrition, whole egg consumption, primary school students, growth, microbiome

## Abstract

Protein-energy malnutrition still impacts children’s growth and development. We investigated the prolonged effects of egg supplementation on growth and microbiota in primary school children. For this study, 8–14-year-old students (51.5% F) in six rural schools in Thailand were randomly assigned into three groups: (1) whole egg (WE), consuming 10 additional eggs/week (*n* = 238) (*n* = 238); (2) protein substitute (PS), consuming yolk-free egg substitutes equivalent to 10 eggs/week (*n* = 200); and (3) control group (C, (*n* = 197)). The outcomes were measured at week 0, 14, and 35. At the baseline, 17% of the students were underweight, 18% were stunted, and 13% were wasted. At week 35, compared to the C group the weight and height difference increased significantly in the WE group (3.6 ± 23.5 kg, *p* < 0.001; 5.1 ± 23.2 cm, *p* < 0.001). No significant differences in weight or height were observed between the PS and C groups. Significant decreases in atherogenic lipoproteins were observed in the WE, but not in PS group. HDL-cholesterol tended to increase in the WE group (0.02 ± 0.59 mmol/L, *ns*). The bacterial diversity was similar among the groups. The relative abundance of *Bifidobacterium* increased by 1.28-fold in the WE group compared to the baseline and differential abundance analysis which indicated that *Lachnospira* increased and *Varibaculum* decreased significantly. In conclusion, prolonged whole egg supplementation is an effective intervention to improve growth, nutritional biomarkers, and gut microbiota with unaltered adverse effects on blood lipoproteins.

## 1. Introduction

Protein-energy malnutrition (PEM) is still a major nutritional problem in the world. It has repercussions on schoolchildren’s growth and development [1]. Inadequate protein intake results in reduced growth and an immune system that is susceptible to disease and infection in early life, and also affects school performance and intelligence status [2], particularly among vulnerable groups [3,4]. Recent data showed that 24.7% of children in Southeast Asian countries were malnourished [5], many of whom lived in households with insecure incomes. School closures led to the disruption of the free school lunch program, exposing millions of children to food insecurity [4]. Our preliminary survey of students in this study in 2021 after the COVID-19 pandemic showed that financial difficulties caused by the lockdown forced families to choose low quality food choices, exacerbating severe malnutrition and disparity in many societies [4].

In Thailand, the government has provided free lunch and milk every school day for primary school children since 1993 and malnutrition has improved over time [6]. However, the recent Thailand National Health Examination Survey showed that about 400,000 (3.5%) Thai children were stunted, while 470,000 (4.1%) were still underweight. In contrast, the prevalence of overnutrition in children has increased and is associated with the early onset of noncommunicable adult chronic diseases [7]. This double burden of malnutrition can be caused by the imbalance of macronutrients and micronutrient intake, particularly vitamin A, iron, vitamin D, and calcium [6,8]. Eggs are a common food around the world that provides approximately 150 kcal/100 g, >50% of adequate intake of critical micronutrients, and high-quality protein, and are more affordable than other animal-derived foods [9]. Eggs are a rich source of choline [10], which plays an integral role in neurotransmitters, cell membrane signaling, and lipid metabolism [11,12]. Recent evidence suggests that the early introduction of one egg per day for six months markedly improved growth in young children [13]. Eggs have been shown to improve growth, as well as reduce wasting and acute malnutrition [14].

Malnutrition has been associated with intestinal dysbiosis [15] by altering the healthy and pathogenic microbiota that efficiently processes foods or produces vitamins. These changes can impact the healthy mucosal immune system. Alterations in the composition of the gut microbiota have been observed in cardiovascular disease (CVD) and malnutrition [16]. 

For example, the number of species in the *Proteobacteria* phylum increases in malnourished infants, while the number of species in the phyla *Bifidobacterium* and *Lactobacillus* decreases [17]. However, recent short-term studies in people revealed that the microbiota is not modified after 4 weeks of egg consumption. Liu et al. showed in a novel but extensive 2-week intervention that it altered vascular function, namely flow-mediated dilation, brachial-ankle pulse wave velocity, and gut microbial function; yet the clear mechanism remains elucidated [18,19]. Therefore, egg consumption may not only help address malnutrition, but may also ameliorate problems with vascular and intestinal function related to alterations in the gut microbiota [19]. Although the short-term benefits of egg supplementation may have been demonstrated, there is considerable controversy regarding its long-term consequences and the underlying mechanism by which egg consumption modifies dysbiosis [11,14]. Therefore, we investigated the effects of prolonged egg supplementation on growth, blood biochemical indices, and gut microbiome in school-aged Thai children.

## 2. Materials and Methods

### 2.1. Study Design and Setting

This cluster randomized controlled trial with parallel design was conducted at six rural primary schools in Nakhon Pathom (Central), Chachoengsao, Chon Buri (Eastern), and Ratchaburi (Western) in Thailand from May 2019 to March 2020. This study was aimed at rural schools where the prevalence of malnutrition was still problematic. The school locations were considered rural areas due to the low population density and no franchise convenience stores within a 10-kilometer radius. We chose rural schools where >10% of all students were underweight based on the weight-for-age (W/A) measurements. The study protocol was approved by the Institutional Review Board of Siriraj Hospital, Mahidol University (COA No. Si 322/2017). Written informed consent was obtained from the parents or legal guardians of the participating children prior to starting the study. This clinical trial was registered with Clinicaltrials.gov (Protocol NCT04896996). This study followed the Consolidated Standards of Reporting Trials (CONSORT) guideline for cluster randomized trials [20].

### 2.2. Sample Size Calculation

The sample size was calculated based on the ability to match the participants in three groups. The effect size of 0.1 for the significant comparison differences between many means was estimated by Cohen, D. A two-tailed significance level of 0.05 and 80% power was used to calculate the sample size for repeated measures ANOVA between factors using G-Power version 3.1. 

### 2.3. Participants and Intervention

**Figure 1:**
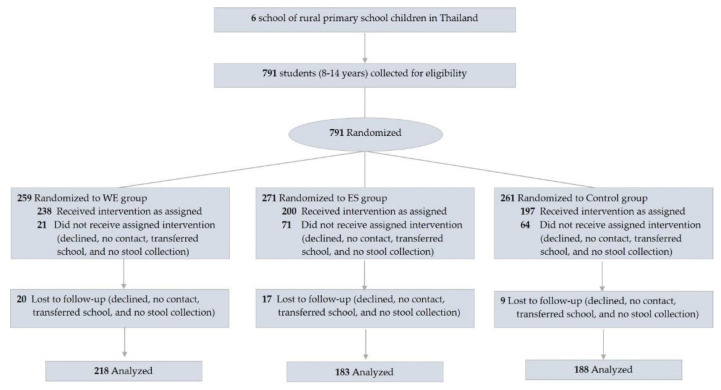
Flow diagram of participants. Abbreviations; WE = whole egg group; PS = protein substitute group.

We recruited students from six rural primary schools and the eligibility criteria included students ages 8–14 years. The participants were excluded if they had an egg allergy. The trial profile is presented in Figure 1. All participants in each school were recruited and randomly assigned to three groups based on the weight-for-age criteria to ensure that all groups were homogeneous: (1) whole egg (WE)—consumed 10 additional whole chicken eggs/week, (2) protein substitute (PS)—consumed a yolk-free egg substitute equivalent to 10 eggs/week, and (3) control group. A cluster randomization was chosen: each classroom in each school was assigned to a group in one of the three groups to reduce group confusion and maintain group compliance. All six schools were asked to prepare the same school lunch menus if possible to standardize the calories and nutritional composition of the meals according to the national school lunch program [6]. 

Before conducting the intervention, all participants were asked to maintain their usual consumption of eggs and dietary cholesterol for four weeks (washout period [week-4]). Participants who were randomized to an intervention (WE and PS) continued their usual dietary habits. The intervention was delivered individually to each classroom at their general lunch time. The WE group received cycle ready-to-eat commercial menu items (S.W. Foodtech., Co., Ltd., Bangkok, Thailand) such as hard-boiled whole eggs, scrambled eggs, stewed eggs, omelets, etc., while the PS group received ready-to-eat commercial menu items such as hard-boiled egg whites or chicken sausages. On average, WE participants received 800 to 850 kcal/d, 2100 to 2260 mg of dietary cholesterol, and 70 to 80 g of protein, while PS participants received 810 to 850 kcal/d, 50 to 220 mg of dietary cholesterol, and 70 to 80 g of protein during the 5 school days. The participants in the control group received standard school lunches according to the Thai school lunch program. No group received additional meals or supplementation on the weekends. The participants recruited in this study were followed up at the baseline, 14 weeks, and 35 weeks. 

### 2.4. Diet Assessment

The participants were invited to participate in semi-structured face-to-face food recall and validated questionnaires with dietitians three times during the study period. The behavior and dietary intake of the children were obtained from a 3-day dietary record [21] to standardize calories and nutritional composition. The energy and nutrient intakes reported in each recall were summed to estimate the observed intakes of complementary feeding. The micronutrients and macronutrients were controlled by the Thai school lunch program (NECTEC), Pathumthani, Thailand) to maintain homogeneity among the schools. Finally, energy intake and nutrients were calculated using INMUCAL–Nutrient Software version 4.0 (INMU), Nakhon Pathom, Thailand.

### 2.5. Outcomes

#### 2.5.1. Anthropometric Measurements

The body weight (BW) and height (HT) were measured (Tanita HD-395, Tanita Corporation, Tokyo, Japan and Institute of Nutrition, Mahidol University [INMU], Thailand). The BW and HT data were converted into percentiles for W/A, height for age (H/A) and weight for height (W/H) using the Thai Growth program software (version 1.05, INMU, Nakhon Pathom, Thailand) [22]. Furthermore, subpopulations have also been characterized according to nutritional status, including underweight, stunting, and wasting, which were defined as Z < −1.5 standard deviations (SDs).

#### 2.5.2. Blood Test

Fasting blood samples were taken for DNA extraction (Supplement Method S1 and Supplement Method S2) and to evaluate hematology (hemoglobin (Hb), hematocrit (Ht), mean corpuscular volume (MCV), transferrin, prealbumin, albumin, fasting blood sugar (FBS), total cholesterol (TC), triglyceride (TG), HDL-cholesterol, LDL-cholesterol, vitamin D, and insulin-like growth factor 1 (IGF-1) were quantified in an accredited clinical laboratory (Siriraj Hospital, Bangkok, Thailand). 

#### 2.5.3. Gut Microbiota Analysis

In total, 15 g of feces were randomly collected in 25% of the participants. Microbial DNA was isolated from 250 mg of feces using a QIAamp PowerFecal Pro DNA Kit (QIAGEN, Hilden, Germany). The samples were sent to the Centre d’expertise et de Services Génome Québec (Génome Québec, Montréal, Canada) for 16S rRNA sequencing. The V4 region of the 16S rRNA gene was amplified using primer 515F–806R, reverse-barcoded: GTGCCAGCMGCCGCGGTAA/ GGACTACHVGGGTWTCTAAT, according to the manufacturer’s protocols. AmpliconSeq sequencing was performed on the NovaSeq platform (Génome Québec, Montréal, Canada) (detail in Supplement Method S3).

### 2.6. Statistical Analysis

Prespecified analyses were performed in three subgroups, as defined by characteristics at randomization: age, sex, W/A, H/A, and W/H. Continuous variables were expressed as mean ± SD and discrete variables as percentages. ANOVA and chi-square tests were used to assess the demographic characteristics and anthropometric data. For repeated measurements, the generalized estimating equation (GEE) was used to determine the effects of group and time for the parameters measured at the baseline, week 14, and week 35. Parameters with only two time points were analyzed using paired *t*-tests to test. GEE was used to determine the differences in absolute changes in dependent variables between the groups. Significant differences were defined as a *p*-value less than 0.05. Statistical analyses were performed using STATA version 17.0 (Stata Corporation, College Station, TX, USA). The gut microbiome used the NovaSeq 6000 platform (Génome Québec, Montréal, Canada) and the sequence reads were processed using QIIME2 version 2021.4 (details in Supplement Method S3).

## 3. Results

### 3.1. Participants

Table 1 represents the baseline characteristics of 635 participants aged 9.8 ± 1.4 years of age. Approximately 12–21% of the participants were underweight and 15–22% were stunted; in contrast, the proportion of overweight and obese participants was over 12% and 6%, respectively, and 70% had low prealbumin levels and low vitamin D levels (Table 1). These results indicate that about a third of this population faced malnutrition of macronutrients or micronutrients. The loss of follow-up was 46 participants (7%) due to illness, relocation, blood draw problems, or personal reasons (Figure 1). No significant differences were observed in the overall mean dietary energy intake and macronutrients, including carbohydrates, protein, fat, and fiber, except cholesterol, between the groups during the study period (Supplement Table S1). Significant differences in the cholesterol levels (mg/day) were observed in the WE (368.5 ± 92.4 mg/day) as compared to the PS (230.3 ± 62.6 mg/day) and control group (236.9 ± 65.2 mg/day), (*p* < 0.001). 

### 3.2. Outcome

#### 3.2.1. Whole Egg Consumption Improved Growth 

**Figure 2:**
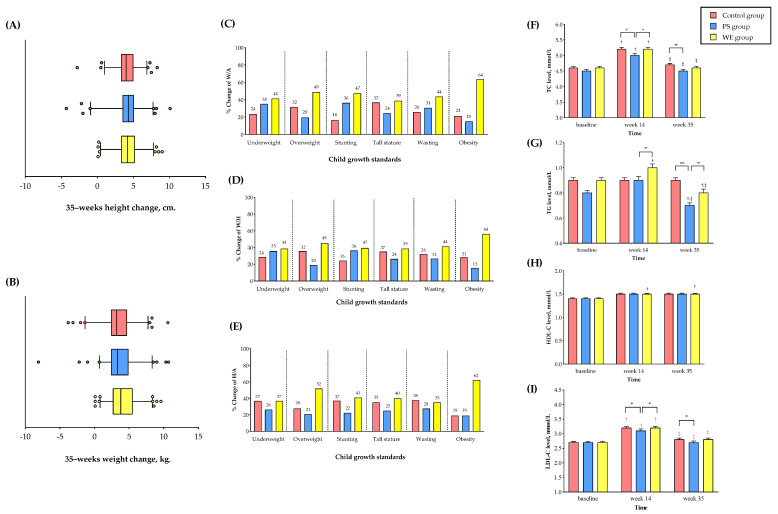
Anthropometrics and plasma lipid levels change in study group. (**A**) Mean of changes in height. (**B**) Mean of changes in weight. (**C**) Percentage of W/A change. (**D**) Percentage of H/A change. (**E**) Percentage of W/H change. (**F**) TC level. (**G**) TG level. (**H**) LDL-C level and (**I**) HDL-C level. The bar graph represents the mean. The error bar indicates the standard error of the mean (SEM). * The statistical significance between the group at *p* < 0.05. ** The statistical significance between the group at *p* < 0.01. *** The statistical significance between the group at *p* < 0.001. † The statistical significance within the group compared to the baseline. ‡ The statistical significance within the group compared to week 14. Abbreviations; PS = protein substitute group; WE = whole egg group; TC = total cholesterol; TG = triglyceride; LDL-C = low-density lipoprotein cholesterol; HDL-C = high-density lipoprotein cholesterol. 2–3.

At week 35, the child growth and malnutrition improved markedly in the WE and PS compared to the C group in almost all anthropometric measures (Table 2 and Supplement Table S2). We observed significant increases in BW and HT in the WE compared to the PS and C group beginning at week 14 and noticeably at week 35. The participants in the WE markedly gained a mean of 21.7 ± 13.5% (4.4 ± 13.7 kg), while participants in the PS and C groups gained a mean of 20.9 ± 15.2% (3.6 ± 13.5 kg) and 19.5 ± 12.4% (3.6 ± 13.3 kg), respectively (WE vs. PS, *p* < 0.001; WE vs. C group, *p* < 0.001). The HT in WE increased by 24.6 ± 8.5% (6.9 ± 13.8 cm), while HT in the PS and C group increased by 22.7 ± 9.7% (3.7 ± 13.6 cm) and 21.6 ± 9.3% (3.4 ± 13.5 cm), respectively (WE vs. PS, *p* < 0.001; WE vs. C group, *p* < 0.001, [Figure 2A,B]). The increase in WE was significantly higher than the reference value recommended by the WHO for children in that age group. No significant differences in BW or HT were observed between the PS and C group after the intervention. In a subpopulation analysis (Figure 2C–E), a higher proportion of participants in the WE than in the PS and C group dramatically improved underweight, stunting, and wasting by 37–41%, 39–47%, and 35–44% (vs. PS [26–36%, 22–36%, and 27–31%] and C [24–37%, 16–37%, and 26–38%]), respectively. Furthermore, children who were overweight, obese, or with a tall stature grew more in both WE and PS than in the C group. WE had a greater improvement in H/A and W/A while PS had a remarkable improvement in BW but not in HT. In brief, child growth and malnutrition markedly improved in prolonged egg supple-mentation.

#### 3.2.2. Plasma Protein

At the baseline, the prealbumin levels < 2.91 μmol/L, as a sensitive indicator of low nutritional status, were found in 5%, 6%, and 6% in the WE, PS and C groups, respectively. The plasma concentrations of both prealbumins increased significantly by 0.24 μmol/L (95% CI, 0.12 to 0.35) in WE compared to the PS and C groups at week 14 and 35 (*p* < 0.001 [Table 2 and Supplement Table S3]).

#### 3.2.3. Cardiometabolic Variables

TC, TG, and HDL levels markedly increased at week 14 compared to the baseline in all groups (*p* < 0.05), while the HDL levels increased significantly only in the WE group but not in the PS and C groups at week 14. Subsequently, at week 35, the TC levels returned to similar levels in all groups compared to the baseline (*ns*), while the TG levels showed a marked decrease in the PS and WE groups but not in the C group, compared to the baseline and week 14 (*p* < 0.05). Surprisingly, the HDL levels increased in the WE group at week 35 (0.08 mmol/L (95% CI, 0.03 to 0.13 [*p* = 0.001]). No significant differences in LDL-C concentration were observed in all groups. However, the mean HDL-C concentration at week 35 had trend increases in the WE group (1.48 ± 0.21 mmol/L) as compared to PS (1.46 ± 0.26 mmol/L) and the C group (1.47 ± 0.26 mmol/L) (WE vs. PS, *p* = 0.410; WE vs. C, *p* = 0.510) shown in Table 2, Figure 2F–I, and Supplement Table S3. Therefore, prolonged egg supplementation modestly improved the lipid levels. 

#### 3.2.4. Gut Microbiota

**Figure 3:**
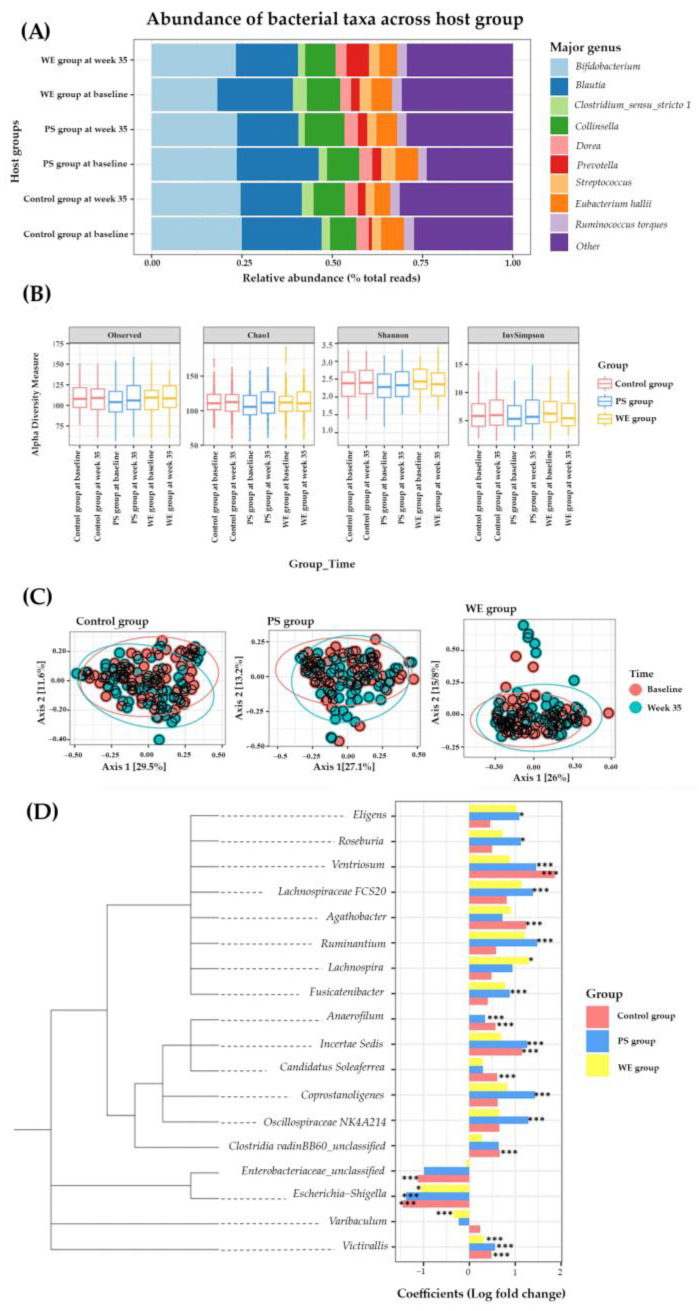
Microbiome change in study group. (**A**) Taxonomy classification. (**B**) Alpha diversity observed in different time points among the host group. (**C**) Multidimensional scaling plot of beta diversity described by Permutational multivariate analysis of variance (PERMANOVA) and the Bray-Curtis dissimilarity measure. (**D**) Differential abundances of bacteria and data are represented by effect size (log fold change of T3/T1) and 95% confidence interval bars (two-sided; FDR adjusted) derived from the ANCOM-BC model. All effect sizes with adjusted *p* < 0.05 are indicated, * *p* < 0.05; *** *p* < 0.001 of significance. Abbreviations; PS = protein substitute group; WE = whole egg group.

A total of 455658 ASVs were detected, corresponding to 2 kingdoms, 29 phyla, 61 classes, 137 orders, 233 families, and 519 genera. Of the 9 genera with the highest abundance in the host group (Figure 3A), there was a significant change in the relative abundance between the baseline and week 35 in WE. The *Bifidobacterium*, found to have a positive effect on the child growth in undernourished children [23], increased up to 1.28 times and *Prevotella* increased 2.63 times and 2.68 times in the WE and C groups, respectively. After egg supplementation in WE, *Prevotella* increased, as reported in an earlier study [24]. Both the alpha or beta bacterial diversity in the WE, PS, and C groups did not significantly change (Figure 3B,C). The genera with higher abundances after supplementation represent a positive direction in the bar graph. In contrast, the genera with lower abundances after supplementation were represented in a negative direction. The abundance of *Agathobacter*, *Candidatus Soleaferrea*, and *Clostridia vadinBB60* was significantly increased in control group. *Enterobacteriaceae* decreased significantly in the control group. Furthermore, the abundance of genera of *Eubacterium Ventriosum*, *Anaerofilum*, and *Incertae Sedis* increased significantly in the control and PS groups (Figure 3D). These results indicated that prolonged egg supplementation promoted healthy gut microbiota.

## 4. Discussion

This randomized controlled trial (RCT) was the first long-term intervention that provided 2 additional whole eggs per school day for 35 weeks, beginning in the first semester and continuing through the second semester in multiple regions of Thailand. We confirmed that this had a positive biological impact on adolescent growth, particularly improving stunting and underweight. This intervention was associated with improved biomarkers, including lipoproteins, microbiota, and healthy dietary patterns in children.

The World Health Organization (WHO) reported a 22.9% prevalence of stunting (H/A Z < −2SD from the median of WHO child growth standards) among children under 5 years of age and a trend in child malnutrition that will be greater than 10 to 50% in Africa, the eastern Mediterranean, and Southeast Asia, including Thailand [25]. We observed that more than 10% of rural primary school children were underweight, stunted or wasted, had low vitamin D levels, low prealbumin levels, or were anemic. These conditions involved an inadequate intake of macronutrients and micronutrients. Our results showed that additional egg consumption may influence healthier dietary patterns. In Thailand, eggs are often eaten with rice, a filling meal that can reduce the need for snacks and desserts. In fact, a previous study in U.S. children showed that egg consumption was significantly associated with higher amounts of several nutrients, including protein, total and saturated fat, alpha-linolenic acid, DHA, lutein + zeaxanthin, choline, potassium, phosphorus, selenium, riboflavin, vitamin D, vitamin A, and vitamin E [26]. Similarly, a cross-sectional survey in the U.S. reported that eggs and foods containing eggs can be an important part of a healthy dietary pattern when balanced with other foods rich in nutrients [27]. Currently, in the post-COVID-19 pandemic, the world is facing socioeconomic inequality, which can lead to starvation and malnutrition. Many low-cost commercial foods are high in calories; on the contrary, they often have poor nutrient profiles.

This finding confirms an RCT that egg consumption significantly improved growth in young children [13]. In Ecuador, supplementation with 1 egg per day in infants for 6 months was reported to have reduced stunting by 47% and increased linear growth by 0.63 length-for-age Z (LAZ) [28]. Mosites et al. showed that in western Kenya the height gain of the child was associated with the consumption of milk and eggs [29] and that an egg was considered a reference food, comparable to breast milk. An egg white is made up of albumin protein—related to muscle mass gain, cell regeneration, and the maintenance of immunity [30]. However, egg yolks also have protein in their composition, as well as vitamin A, vitamin E, vitamin D, and, the most expressive of this complex, choline. Choline is a nutrient that plays a role in human metabolism and cell membrane structure, and acts on the transmission of nerve impulses [31]. During pregnancy and lactation, it is essential for the development of the nervous system of the fetus [32,33]. In older adults, Liu et al. suggest that choline plays a role in maintaining the nerve impulse circuit, preventing age-related cognitive decline, and maintaining memory [34]. The egg is one of the few foods that has vitamin D (fat-soluble vitamins), responsible for the deposition of bone calcium and the mineralization of the skeleton [35]. It also has vitamins A and E, which have an antioxidant action. Moreover, the egg has in its composition several minerals such as calcium, phosphorus, iron, magnesium, manganese, zinc, copper, and selenium - found in the egg and which meet 50% of the needs of adults and children [36]. 

Furthermore, we found that egg supplementation improved the blood lipid profiles, including HDL-C levels [37]. Similarly, daily egg consumption promotes HDL lipid composition and function [38]. Fernandez et al. reported that eating whole eggs increases the size of HDL lipoprotein particles and increases the activity of lecithin-cholesterol acyltransferase (LCAT) [39]. The yolk has mono- and polyunsaturated fatty acids, considered good fats for heart health, a small amount of saturated fat, and has cholesterol in its composition, which has already been proven by numerous studies not to be associated with an increased risk of cardiovascular disease and stroke [40,41]. Recently, U.S. cohort studies and meta-analysis data showed that moderate egg consumption (up to one egg per day) is not associated with a potentially lower risk of cardiovascular disease in Asian populations [41].

Regarding the structure of the gut microbiome after whole egg supplementation, we observed increased levels of *Bifidobacterium* in the WE group. *Bifidobacterium* is a human milk oligosaccharide (HMO) used by bacteria [42]. They are considered to have health-promoting benefits in humans [43]. These microbes produce a variety of useful metabolites, which benefit the host’s immune system [42]. On the contrary, a decrease in this microbiota has been associated with a high incidence of diseases, such as irritable bowel syndrome (IBS) [44]. In Thai children, the abundance of *Bifidobacterium* is negatively correlated with the consumption of fish and beef [45]. In our study, the abundance of *Lachnospira* was significantly higher after WE supplementation. *Lachnospira* are anaerobic, fermentative, and chemoorganotrophic [46]. Normally, this genus is well known as one of the SCFA producers throughout the whole grain fermenter [47]. Vanegas et al. reported that short-term supplementation of whole or refined grains increased the abundance of *Lachnospira* significantly [48]. Our results showed that the abundance of *Varibaculum* was significantly lower after whole egg supplementation. Furthermore, there is little evidence of the relationship between *Varibaculum* and host health at the genus level. Kang et al. reported that the abundance of *Varibaculum* was significantly higher in patients with invasive cervical cancer (CAN) compared to healthy controls [49].

This research has strengths which suggest that its findings may have important implications for public policy. First, this is a large-scale, one-year randomized controlled trial. We collected data from rural schoolchildren, including central, eastern, and western Thailand, homogenized by geographical and food patterns. Second, we used tools for the evaluation of food intake to achieve a high level of precision of nutrition data. Third, this study showed an important verified discovery that the fight against malnutrition, especially in low- and middle-income communities, could be achieved by using locally available high-quality proteins such as eggs, milk, and chicken. This also impacts healthier food choices and children’s behavior. However, there are some limitations of this study. First, whole egg consumption in the protein substitute and control groups on weekends and during school breaks is difficult to control. Second, the whole egg group and the protein substitute group had at least one secondary school class, suggesting that these may be confounding variables for anthropometric analysis. 

## 5. Conclusions

In conclusion, long-term whole egg supplementation is a feasible, low-cost, and effective intervention to significantly increase growth and improve important biomarkers in young school-age children without adverse effects on blood cholesterol levels. It also promotes intestinal microbial diversity by maintaining an intestinal microbiota composition that benefits health. More information is needed on the mechanistic effects of egg consumption on gut microbiota and growth.

## Figures and Tables

**Table 1:** Baseline characteristics of participants.

Variables	Control [*n* = 197]	PS [*n* = 200]	WE [*n* = 238]
	*n* (%)	*n* (%)	*n* (%)
Age, mean (SD), year	9.2 (0.1)	9.5 (0.1)	9.6 (0.3)
Sex			
Male	103 (52)	97 (49)	108 (45)
Female	94 (48)	103 (51)	130 (55)
Career of parents			
Government officials	5 (3)	9 (6)	13 (6)
Self-employment	23 (13)	27 (16)	38 (18)
Agriculturist	55 (32)	45 (27)	59 (28)
Company employee	10 (6)	22 (13)	28 (13)
Unemployed	9 (5)	12 (7)	20 (9)
Others (i.e., contractor)	71 (41)	52 (31)	55 (26)
Weight, mean (SD), kg	31.6 (9.5)	31.6 (8.1)	32.1 (9.4)
Height, mean (SD), cm	137.1 (8.8)	137.8 (9.3)	138.7 (9.0)
W/A, mean (SD), percentile	103.7 (27.9)	100.3 (22.8)	103.4 (26.7)
Underweight	24 (12)	42 (21)	40 (17)
Overweight	19 (10)	11 (6)	12 (5)
H/A, mean (SD), percentile	100.1 (4.5)	100.1 (4.4)	100.3 (5.2)
Stunted	29 (15)	44 (22)	41 (17)
W/H, mean (SD), percentile	102.3 (18.6)	99.2 (14.7)	102.2 (19.4)
Wasted	24 (12)	37 (19)	21 (9)
Obese	25 (13)	36 (18)	17 (7)
Blood pressure, mean (SD), mm Hg			
Systolic	103.2 (9.1)	103.7 (9.1)	104.2 (9.8)
Diastolic	69.7 (5.6)	70.7 (5.4)	70.6 (5.9)
Hemoglobin, mean (SD), mmol/L	7.9 (0.6)	8.0 (0.6)	8.0 (0.7)
<7.13	21 (11)	19 (10)	24 (10)
Hematocrit, mean (SD), %	39.18 (2.9)	39.50 (2.8)	39.73 (3.3)
<35	17 (9)	20 (10)	24 (10)
MCV, mean (SD), fL	78.2 (5.6)	78.5 (4.9)	78.4 (6.3)
<80	125 (91)	126 (63)	152 (64)
Fasting blood sugar, mean (SD), mmol/L	4.8 (0.5)	5.0 (0.5)	4.8 (0.5)
Transferrin, mean (SD), g/L	2.6 (0.3)	2.6 (0.3)	2.6 (0.3)
Prealbumin, mean (SD), μmol/L	3.8 (0.6)	3.9 (0.6)	3.9 (0.6)
<2.91	12 (6)	12 (6)	13 (6)
Albumin, mean (SD), g/L	43.7 (2.3)	43.6 (2.1)	43.9 (2.1)
Blood lipid level, mean (SD), mmol/L			
TC	4.6 (0.6)	4.5 (0.7)	4.6 (0.7)
TG	0.9 (0.3)	0.8 (0.3)	0.9 (0.3)
HDL-C	1.4 (0.3)	1.4 (0.3)	1.4 (0.3)
LDL-C	2.7 (0.5)	2.7 (0.6)	2.7 (0.6)
Vitamin D, mean (SD), nmol/L	70.6 (15.7)	62.2 (18.7)	65.9 (16.5
<74.88, % (95% CI)	60 (57.1–63.2)	75 (71.0–78.0)	75 (72.0–77.0)
49.92–72.38, % (95% CI)	58 (54.1–61.2)	53 (51.9–56.7)	58 (53.9–60.1)
<49.92, % (95% CI)	2 (1.0–3.3)	17 (14.8–18.8)	23 (20.9–25.5)
IGF-1, mean (SD), nmol/L	28.7 (14.5)	34.7 (16.5)	36.0 (18.0)

Abbreviations: PS, protein substitute group; WE, whole egg group; W/A, weight for age; H/A, height for age; W/H, weight for height; MCV, mean corpuscular volume; TC, total cholesterol; TG, triglyceride; HDL-C, high-density lipoprotein cholesterol; LDL-C, low-density lipoprotein cholesterol; 95% CI, 95% Confidence Interval; IGF-1, insulin-like growth factor 1.

**Table 2:** The 14 and 35 weeks change estimates for anthropometric and biochemical indices of participants.

Variables	Control [*n* = 197]	PS [*n* = 200]	WE [*n* = 238]	*p*-Value ^a^
	Mean within Group Difference (95% CI) ^b^	Mean within Group Difference (95% CI) ^c^	Mean within Group Difference (95% CI) ^d^	
H/A, percentile				
week 14	+0.25 (−0.68–1.18)	+0.01 (−0.93–0.95)	+0.33 (−0.51–1.17)	0.714
week 35	+0.49 (−0.44–1.41)	+0.18 (−0.76–1.11)	+0.50 (−0.36–1.35)	0.412
W/A, percentile				
week 14	−1.16 (−6.41–4.09)	+0.53 (−4.71–5.77)	+3.01 (−1.82–7.84)	0.402
week 35	+2.21 (−3.03–7.46)	+4.53 (−0.73–9.78)	+5.00 (0.13–9.87)	0.063
W/H, percentile				
week 14	−0.45 (−4.01–3.11)	+1.02 (−2.60–4.65)	+0.56 (−2.71–3.82)	0.685
week 35	+1.22 (−2.33–4.77)	+2.99 (−0.65–6.62)	+2.98 (−0.31–6.26)	0.415
Height, cm				
week 14	+0.92 (−0.96–2.80)	+1.10 (−0.77–2.97)	+4.07 (2.32–5.83)	<0.001
week 35	+3.41 (1.53–5.30)	+3.72 (1.84–5.61)	+6.91 (5.16–8.67)	<0.001
Weight, kg				
week 14	+0.91 (−0.95–2.77)	+1.02 (−0.83–2.88)	+1.62 (−0.11–3.35)	0.001
week 35	+3.58 (1.71–5.44)	+3.62 (1.75–5.49)	+4.39 (2.65–6.13)	<0.001
Subpopulation				
Underweight				
Height, cm				
week 14	+0.19 (−2.52–2.90)	+0.64 (−2.13–3.38)	+1.62 (−1.33–4.29)	0.010
week 35	+1.20 (−1.77–4.18)	+3.87 (1.02–6.69)	+2.61 (−0.61–5.54)	0.030
Weight, kg				
week 14	+0.59 (−0.80–1.97)	+0.21 (−1.22–1.65)	+0.94 (−0.45–2.32)	0.024
week 35	+1.49 (−0.04–3.01)	+2.17 (0.69–3.64)	+1.17 (−0.32–2.67)	0.378
Overweight				
Height, cm				
week 14	+1.56 (−1.57–4.69)	+1.57 (−1.63–4.74)	+5.04 (1.95–7.86)	0.029
week 35	+2.08 (−1.04–5.21)	+4.05 (0.89–7.19)	+7.13 (4.06–9.92)	0.006
Weight, kg				
week 14	+0.80 (−3.33–4.94)	+1.20 (1.01–5.41)	+1.61 (1.92–5.14)	0.015
week 35	+2.92 (−1.15–6.99)	+4.04 (1.13–8.20)	+4.94 (1.42–8.45)	0.043
Stunted				
Height, cm				
week 14	+2.67 (−5.81–11.14)	+0.40 (−6.17–6.97)	+3.42 (−4.51–11.35)	0.022
week 35	+2.55 (−6.93–12.03)	+2.50 (−4.07–9.07)	+7.63 (0.04–15.21)	0.010
Weight, kg				
week 14	+0.58 (−6.62–7.79)	+0.00 (−5.97–5.97)	+1.20 (−5.13–7.53)	0.437
week 35	−0.20 (−8.37–7.97)	+5.66 (−0.31–11.63)	+2.58 (−3.13–8.29)	0.352
Wasted				
Height, cm				
week 14	+0.61 (−3.43–4.64)	+1.00 (−2.55–4.52)	+5.12 (1.11–8.78)	0.344
week 35	+2.31 (−1.90–6.53)	+3.58 (−0.20–7.34)	+7.85 (3.50–11.80)	0.501
Weight, kg				
week 14	+1.54 (−0.92–4.00)	+0.26 (−1.97–2.48)	+1.25 (−0.97–3.47)	0.661
week 35	+2.80 (0.22–5.38)	+1.51 (−0.86–3.88)	+2.17 (−0.22–4.55)	0.489
Obesity				
Height, cm				
week 14	+1.62 (−1.93–5.18)	+0.70 (−2.99–4.36)	+4.06 (0.74–7.08)	0.041
week 35	+2.06 (−1.33–5.44)	+3.66 (0.07–7.22)	+6.14 (2.86–9.12)	0.026
Weight, kg				
week 14	+2.42 (−2.78–7.62)	+1.08 (−4.34–6.51)	+1.81 (−2.45–6.07)	0.009
week 35	+2.80 (−2.08–7.68)	+3.71 (−1.57–8.98)	+6.13 (1.85–10.40)	0.032
Transferrin, g/L				
week 14	+0.07 (0.01–0.12)	+0.10 (0.04–0.16)	+0.06 (0.01–0.12)	0.033
week 35	+0.15 (0.10–0.21)	+0.15 (0.09–0.20)	+0.16 (0.11–0.21)	0.008
Prealbumin, μmol/L				
week 14	+0.02 (−0.10–0.14)	+0.08 (−0.04–0.20)	+0.16 (0.04–0.27)	<0.001
week 35	+0.05 (−0.08–0.17)	−0.01 (−0.13–0.11)	+0.24 (0.12–0.35)	<0.001
Prealbumin < 2.91 μmol/L (%)	5.3 (4.8–5.9)	5.3 (4.1–5.6)	4.4 (3.9–5.2)	0.712
Albumin, g/L				
week 14	−1.13 (−1.57–0.70)	−0.93 (−1.36–0.50)	−0.34 (−0.76–0.07)	0.001
week 35	−0.53 (−0.96–0.10)	−0.37 (−0.81–0.06)	−0.19 (−0.60–0.22)	<0.001
Hemoglobin, mmol/L				
week 14	+0.08 (−0.04–0.20)	−0.02 (−0.14–0.10)	+0.11 (0.00–0.22)	0.042
week 35	−0.15 (−0.27–0.03)	−0.19 (−0.31–0.06)	−0.10 (−0.21–0.01)	0.031
Hematocrit, %				
week 14	+0.39 (−0.15–0.93)	+0.12 (−0.42–0.66)	+0.60 (0.11–1.10)	0.037
week 35	−1.89 (−2.43–1.36)	−2.10 (−2.64–1.56)	−1.79 (−2.29–1.30)	0.045
MCV, fL				
week 14	−0.16 (−1.28–0.96)	−0.04 (−1.17–1.09)	+0.01 (−1.02–1.04)	0.957
week 35	−0.29 (−1.42–0.84)	−0.30 (−1.44–0.84)	−0.08 (−1.11–0.95)	0.780
MCV < 80 fL (%)	58.4 (56.1–61.6)	58.0 (53.1–60.8)	55.3 (51.9–57.5)	0.485
FBS, mmol/L				
week 14	+0.06 (−0.04–0.12)	+0.02 (−0.04–0.06)	+0.12 (0.04–0.19)	0.648
week 35	+0.27 (0.19–0.35)	+0.16 (−0.02–0.16)	+0.24 (0.17–0.32)	0.385
TC, mmol/L				
week 14	+0.55 (0.41–0.69)	+0.48 (0.34–0.63)	+0.61 (0.48–0.74)	0.046
week 35	+0.11 (−0.03–0.25)	+0.01 (−0.13–0.16)	+0.07 (−0.06–0.20)	0.049
TG, mmol/L				
week 14	+0.04 (−0.02–0.11)	+0.06 (−0.01–0.12)	+0.06 (0.00–0.12)	0.032
week 35	−0.02 (−0.08–0.05)	−0.09 (−0.16–0.03)	−0.08 (−0.14–0.02)	0.046
HDL-C, mmol/L				
week 14	+0.03 (−0.02–0.08)	+0.03 (−0.02–0.08)	+0.06 (0.02–0.11)	0.181
week 35	+0.03 (−0.02–0.08)	+0.04 (−0.01–0.09)	+0.08 (0.03–0.13)	0.427
LDL-C, mmol/L				
week 14	+0.51 (0.38–0.64)	+0.43 (0.29–0.56)	+0.52 (0.40–0.64)	0.031
week 35	+0.12 (−0.01–0.25)	+0.03 (−0.10–0.16)	+0.05 (−0.07–0.17)	0.010

Abbreviations: PS, protein substitute group; WE, whole egg group; H/A, height for age; W/A, weight for age; W/H, weight for height; MCV, mean corpuscular volume; FBS, fasting blood sugar; TC, total cholesterol; TG, triglyceride; HDL-C, high-density lipoprotein cholesterol; LDL-C, low-density lipoprotein cholesterol. ^a^ Statistically significant difference between groups; ^b^ mean difference within the control group 95% CI compared to baseline; ^c^ mean difference within the PS group 95% CI compared to baseline; ^d^ mean difference within the WE group 95% CI compared to baseline.

## Data Availability

Not applicable.

## Supplementary Materials

The following supporting information can be downloaded at: https://www.mdpi.com/article/10.3390/nu15051143/s1, Supplement Method S1: Plasma and serum collection; Supplement Method S2: Human DNA extracted from blood sample; Supplement Method S3: Bacterial DNA extracted from stool sample; Supplement Table S1: Average nutrient intake of participants during the study period; Supplement Table S2: Mean change estimates for anthropometric of participants; Supplement Table S3: Mean change estimates for biochemical indices of participants.
