# Supplementary material for: Prolonged Egg Supplement Advances Growing Child’s Growth and Gut Microbiota
Source: Nutrients. 2023 Feb 24;15(5):1143. doi: 10.3390/nu15051143 (PMC10005095; doi:10.3390/nu15051143)
Supplement: Supplementary file 1 [file nutrients-15-01143-s001.zip › nutrients-2224410-supplementary.pdf]

]Supplement Method S1: Plasma and serum collection

1. Venous blood (6 mL) into an EDTA tube (treated with an anticoagulant) for plasma and into a clotted blood tube for serum.
2. For serum, put sample tube at room temperature for 30 minutes before centrifugation.
3. All plasma and serum were centrifuged at 3,000 rpm at 4 ° C for 10 minutes by centrifugator.
4. Aliquot plasma and serum
5. Storage at -20 ° C (analysis within 1 month) and -80 ° C for study in the future.

Supplement Method S2: Human DNA extracted from blood sample

An Automated Chemagic 360 instrument (CMG-1074, PerkinElmer, Baesweiler, Germany) was used. Briefly, 2 mL of whole blood was collected into a deep well, and 15 µL of protein kinase was added to each well. Preparation of reagents and instruments according to the manufacturer's instructions. The DNA concentration and a purity ratio (acceptable in range 1.8-1.9) were measured using FLUOstar Omega (software version 5.5 R4, BMG LABTECH, Ortenberg, Germany).

Supplement Method S3: Bacterial DNA extracted from stool sample

The QIAamp® PowerFecal® Pro DNA Kit (QIAGEN, Hilden, Germany) was used (following to the manufacturer). In a PowerBead Pro Tube, 250 mg of stool was weighed, and 800 µL of solution CD1. Vortex using PowerLyzer 24 Homogenizer at maximum speed for 10 min. and centrifuged at 15000 g for 1 minute. Pipette 600 µL of the supernatant into a microcentrifuge tube and 200 µL of solution CD2 was added after centrifugation. Transfer 700 µL of supernatant to a microcentrifuge tube and add 600 µL of solution CD3. The lysate (650 µL) was loaded into the MB spin column and centrifuged. The flow-through was discarded, the MB spin column was placed into a collection tube, and 500 µL of EA solution was added. The flow-through was then centrifuged and discarded. Then, 500 µL of solution C5 was added to the MB spin column, centrifuged, and the flow-through discarded. The MB spin column was carefully centrifuged for 2 minutes at 16 000 g. Finally, 50 µL of solution C6 and centrifuge at 15 000 × g for 1 minute. The MB spin column was discarded. DNA is currently ready for downstream applications. Microbial DNA concentration and a purity ratio (acceptable in range 1.8-1.9) were measured using FLUOstar Omega (software version 5.5 R4, BMG LABTECH, Ortenberg, Germany).

The samples were sent to the Centre d'expertise et de Services Génome Québec (Génome Québec, Montréal, Canada) for 16S rRNA sequencing. The gut microbiome study used the NovaSeq 6000 platform and Illumina sequencing by synthesis (SBS) to generate low error rate amplicon data. The analysis focused on V4 of the 16s rRNA genes, where 515F-806R was used as a primer. AmpliconSeq sequencing was performed on the NovaSeq platform (Génome Québec, Montréal, Canada). The raw sequence reads were processed using QIIME2 version 2021.4 and operated according to the standard pipeline recommended by Hall and Beiko. Briefly, the primers were trimmed and sequences with a quality score lower than 30 were filtered and further analyzed by merging the forward and reverse reads. The chimera was defined and removed by testing at least six abundances as potential parents. Taxonomy was classified with at least 97% sequence similarity to the Silva v132 database. The amplicon sequence variant (ASV) with an abundance of less than two sequences was filtered out. Phylogenetic analysis was performed by aligning the ASVs using MAFFT and a tree with FastTree. Finally, alpha and beta diversity and differential abundance were analyzed and visualized using the R package (details were described in Supplement Method 3). We generated a principal coordinate analysis (PCoA) using the weighted UniFrac distances between the first (T1) and the last time point (T3) of each group. The analysis of the composition of microbiomes with bias correction (ANCOM-BC) was applied to analyze the differential abundance in this study. We used pairwise comparisons with false discovery rate (FDR) correction to estimate the differences in taxonomic composition between the time points of each host group. The ASVs classified by taxonomy from the previous step were imported into the R platform. We used the observed ASVs, Chao1 richness, Shannon index, and the InvSimpson index for the alpha diversity estimator. These were used to characterize the diversity of species among groups. PCoA was generated from weighted UniFrac distances for the beta diversity analysis.

Supplement Table S1. Average nutrient intake of participants during the study period				
**Energy/Nutrients**	**Control [n=39]**	**PS [n=41]**	**WE [n=45]**	***p-value***
	**Mean (SD)**	**Mean (SD)**	**Mean (SD)**	
Energy, kcal/day	1029.2 (143.5)	1021.8 (129.3)	1018.0 (126.5)	0.343
Carbohydrate, g/day	124.1 (19.8)	117.9 (17.9)	116.0 (17.0)	0.345
Protein, g/day	41.7 (7.4)	46.6 (6.7)	44.8 (7.0)	0.136
Fat, g/day	40.7 (7.8)	40.4 (6.7)	41.7 (7.0)	0.486
Saturated fatty acids, g/day	11.6 (2.9)	11.8 (2.8)	11.1 (2.8)	0.607
Cholesterol, mg/day	236.9 (65.2)[a]	230.3 (62.6)[a]	368.5 (92.4)[b]	<0.001
Dietary fiber, g/day	4.2 (1.9)	3.7 (1.0)	3.8 (5.4)	0.880
Sodium, mg/day	1559.4 (378.9)	1512.6 (326.6)	1456.1 (308.8)	0.656
Calcium, mg/day	386.8 (111.4)	366.4 (89.8)	377.9 (113.7)	0.923
Iron, mg/day	5.1 (1.1)	5.4 (1.0)	5.6 (1.3)	0.098
Zinc, mg/day	3.0 (0.7)	3.4 (0.6)	3.2 (0.6)	0.174
Vitamin A, RAE μg/day	274.0 (143.0)	281.0 (165.4)	446.0 (291.3)	0.042
Beta-carotene, mcg/day	395.6 (246.5)	383.2 (228.9)	409.4 (240.2)	0.506
Thiamin, mg/day	0.8 (0.3)	1.0 (1.1)	0.8 (0.3)	0.980
Riboflavin, mg/day	0.8 (0.2)	1.0 (0.2)	0.9 (0.2)	0.435
Niacin, mg/day	7.1 (1.7)	7.9 (1.8)	7.2 (1.7)	0.111
Vitamin C, mg/day	22.6 (13.2)	19.1 (12.0)	16.5 (9.3)	0.776

Abbreviation: PS, protein substitute group; WE, whole egg group; RAE, retinol activity equivalents.
[a,b] Values in the same row with the same superscript letters are significantly different among the groups at $p<0.05$.

Supplement Table S2. Mean change estimates for anthropometric of participants				
Variables	Control [n=197]	PS [n=200]	WE [n=238]	*p-value*
	Mean (SD)	Mean (SD)	Mean (SD)	
H/A				
baseline	100.1 (4.5)	100.1 (4.4)	100.3 (5.2)	0.399
week 14	100.4 (4.6)	100.1 (4.4)	100.6 (5.2)	0.714
week 35	100.6 (4.5)	100.3 (4.6)	100.8 (5.1)	0.412
change	+0.5	+0.2	+0.5	
W/A				
baseline	103.7 (27.9)	100.3 (22.7)	103.4 (26.7)	0.317
week 14	102.6 (25.9)	100.9 (24.5)	106.4 (29.2)	0.402
week 35	106.0 (27.0)	104.9 (27.3)	108.4 (30.3)	0.063
change	+2.2	+4.5	+5.0	
W/H				
baseline	102.3 (18.6)	99.2 (14.7)	102.2 (19.4)	0.688
week 14	101.9 (17.9)	100.3 (16.5)	102.7 (19.7)	0.685
week 35	103.5 (18.1)	102.2 (18.0)	105.1 (20.7)	0.415
change	+1.2	+3.0	+3.0	
Height, cm				
baseline	137.1 (8.9)	137.8 (9.3)	138.67 (9.0)	0.358
week 14	138.0 (9.6)[a]	138.9 (10.0)[a]	142.74 (10.0)[b]	<0.001
week 35	140.5 (9.5)[a]	141.5 (10.0)[a]	145.59 (10.4)[b]	<0.001
change	+3.4	+3.7	+6.9	
Weight, kg				
baseline	31.6 (9.6)[a]	31.6 (8.1)[a]	34.3 (9.3)[b]	0.016
week 14	32.5 (9.6)[a]	32.6 (8.8)[a]	35.9 (9.8)[b]	0.001
week 35	35.2 (10.4)[a]	35.2 (9.7)[a]	38.7 (11.0)[b]	<0.001
change	+3.6	+3.6	+4.4	
**Subpopulation**				
Underweight				
Height, cm				
baseline	131.8 (13.50)	133.4 (14.5)	134.4 (13.7)	0.246
week 14	132.0 (13.88)[a]	134.1 (15.4)[ab]	136.0 (15.1)[b]	0.010
week 35	133.0 (15.64)[a]	137.3 (17.8)[b]	137.0 (14.8)[b]	0.030
change	+1.2	+3.9	+2.6	
Weight, kg				
baseline	24.7 (6.7)[a]	25.4 (7.2)[ab]	26.3 (7.7)[b]	0.018
week 14	25.3 (6.3)[a]	25.6 (7.8)[a]	27.2 (8.2)[b]	0.024
week 35	26.2 (8.0)	27.6 (9.1)	27.5 (9.2)	0.378
change	+1.5	+2.2	+1.2	
Overweight				
Height, cm				
baseline	142.8 (14.9)	143.0 (18.9)	143.5 (15.0)	0.757
week 14	144.3 (14.3)[a]	144.6 (18.4)[a]	148.5 (14.9)[b]	0.029
week 35	144.8 (12.3)[a]	147.1 (18.5)[a]	150.6 (14.4)[b]	0.006

change	+2.1	+4.1	+7.1	
Weight, kg				
baseline	46.0 (23.2)^{ab}	42.8 (19.8)^{a}	47.5 (18.0)^{b}	0.020
week 14	46.8 (22.9)^{ab}	44.0 (19.4)^{a}	49.1 (18.7)^{b}	0.015
week 35	48.9 (23.3)^{ab}	46.8 (19.5)^{a}	52.4 (20.0)^{b}	0.043
change	+2.9	+4.0	+4.9	
Stunted				
Height, cm				
baseline	120.0 (35.3)	125.0 (46.7)	127.3 (50.7)	0.277
week 14	122.7 (32.8)^{a}	125.4 (45.3)^{ab}	130.8 (48.5)^{b}	0.022
week 35	122.6 (20.4)^{a}	127.5 (43.5)^{a}	135.0 (36.4)^{b}	0.010
change	+2.6	+2.5	+7.6	
Weight, kg				
baseline	21.8 (17.5)	22.2 (22.5)	25.8 (29.8)	0.445
week 14	22.3 (26.1)	22.2 (24.3)	27.0 (39.3)	0.437
week 35	21.6 (7.7)	27.9 (67.9)	28.4 (33.3)	0.352
change	-0.2	+5.7	+2.6	
Wasted				
Height, cm				
baseline	138.8 (19.5)	137.6 (18.7)	136.4 (17.8)	0.419
week 14	139.5 (21.7)	138.6 (19.3)	141.5 (20.5)	0.344
week 35	141.2 (22.4)	141.2 (20.8)	144.3 (23.1)	0.501
change	+2.3	+3.6	+7.9	
Weight, kg				
baseline	26.4 (12.7)	27.1 (11.4)	27.9 (12.6)	0.392
week 14	27.9 (13.1)	27.4 (11.6)	29.2 (12.6)	0.661
week 35	29.2 (13.5)	28.7 (11.8)	30.1 (14.6)	0.489
change	+2.8	+1.5	+2.2	
Obesity				
Height, cm				
baseline	141.6 (16.3)	139.4 (20.8)	141.0 (15.6)	0.436
week 14	143.2 (16.9)^{ab}	140.1 (20.8)^{a}	145.1 (17.0)^{b}	0.041
week 35	143.7 (15.3)^{a}	143.1 (19.0)^{ab}	147.1 (17.3)^{b}	0.026
change	+2.1	+3.7	+6.1	
Weight, kg				
baseline	47.5 (27.0)	43.1 (26.6)	47.5 (22.6)	0.386
week 14	49.9 (29.0)^{a}	44.2 (25.7)^{b}	49.3 (23.8)^{a}	0.009
week 35	50.3 (26.5)^{ab}	46.8 (24.2)^{b}	53.6 (25.4)^{b}	0.032
change	+2.8	+3.7	+6.1	

Abbreviation: PS, protein substitute group; WE, whole egg group; H/A, height for age; W/A, weight for age; W/H, weight for height. ^{a,b} Values in the same row with the same superscript letters are significantly different among the groups at $p<0.05$.

Supplement Table S3. Mean change estimates for biochemical indices of participants				
**Variables**	**Control [n=197]**	**PS [n=200]**	**WE [n=238]**	***p-value***
	**Mean (SD)**	**Mean (SD)**	**Mean (SD)**	
Transferrin, g/L				
baseline	2.6 (0.3)	2.6 (0.3)	2.6 (0.3)	0.142
week 14	2.6 (0.3)[a]	2.7 (0.3)[b]	2.7 (0.3)[ab]	0.033
week 35	2.7 (0.3)[a]	2.8 (0.3)[ab]	2.8 (0.3)[b]	0.008
change	+0.2	+0.2	+0.2	
Prealbumin, μmol/L				
baseline	3.8 (0.6)	3.9 (0.6)	3.9 (0.6)	0.771
week 14	3.8 (0.6)[a]	3.9 (0.6)[a]	4.1 (0.7)[b]	<0.001
week 35	3.9 (0.6)[a]	3.9 (0.6)[a]	4.2 (0.8)[b]	<0.001
change	+0.1	0.0	+0.2	
Albumin, g/L				
baseline	43.7 (2.3)	43.6 (2.1)	43.9 (2.1)	0.420
week 14	42.6 (1.9)[a]	42.6 (1.9)[a]	43.6 (2.0)[b]	0.001
week 35	43.2 (2.7)[a]	43.2 (2.7)[a]	43.7 (2.4)[b]	<0.001
change	-0.5	-0.4	-0.2	
Hemoglobin, mmol/L				
baseline	7.9 (0.6)	8.0 (0.6)	8.0 (0.7)	0.757
week 14	8.0 (0.5)[ab]	8.0 (0.5)	8.1 (0.7)[b]	0.042
week 35	7.8 (0.6)[a]	8.0 (0.6)[a]	7.9 (0.6)[b]	0.031
change	-0.2	-0.3	-0.1	
Hematocrit, %				
baseline	39.2 (2.9)[a]	39.5 (2.8)[ab]	39.7 (3.3)[b]	0.049
week 14	39.6 (2.2)[a]	39.6 (2.5)[a]	40.3 (3.2)[b]	0.037
week 35	37.3 (2.4)[a]	37.4 (2.4)[a]	37.9 (2.6)[b]	0.045
change	-1.9	-2.1	-1.8	
MCV, fL				
baseline	78.2 (5.6)	78.5 (4.9)	78.4 (6.3)	0.755
week 14	78.0 (5.8)	78.5 (5.2)	78.4 (6.6)	0.957
week 35	77.9 (5.5)	78.2 (5.1)	78.3 (6.5)	0.780
change	-0.3	-0.6	-0.1	
FBS, mmol/L				
baseline	4.8 (0.5)	4.9 (0.5)	4.8 (0.5)	0.234
week 14	4.9 (0.4)	4.9 (0.4)	4.9 (0.6)	0.648
week 35	5.1 (0.4)	5.0 (0.4)	5.1 (0.5)	0.385
change	+0.3	+0.2	+0.3	
TC, mmol/L				
baseline	4.6 (0.6)	4.5 (0.7)	4.6 (0.7)	0.131
week 14	5.2 (0.8)[a]	5.0 (0.8)[b]	5.2 (0.9)[a]	0.046
week 35	4.7 (0.6)[a]	4.5 (0.7)[b]	4.6 (0.8)[ab]	0.049

change	+0.1	+0.0	+0.1	
TG, mmol/L				
baseline	0.9 (0.3)[ab]	0.8 (0.3)[a]	0.9 (0.3)[b]	0.017
week 14	0.9 (0.3)[ab]	0.9 (0.4)[a]	1.0 (0.4)[b]	0.032
week 35	0.9 (0.3)[a]	0.7 (0.3)[b]	0.8 (0.4)[a]	0.046
change	-0.0	-0.1	-0.1	
HDL-C, mmol/L				
baseline	1.4 (0.3)	1.4 (0.3)	1.4 (0.3)	0.604
week 14	1.5 (0.3)	1.5 (0.3)	1.5 (0.2)	0.181
week 35	1.5 (0.3)	1.5 (0.3)	1.5 (0.2)	0.427
change	+0.0	+0.0	+0.1	
LDL-C, mmol/L				
baseline	2.7 (0.5)	2.7 (0.6)	2.7 (0.6)	0.113
week 14	3.2 (0.7)[a]	3.1 (0.8)[b]	3.2 (0.8)[a]	0.031
week 35	2.8 (0.7)[a]	2.7 (0.7)[b]	2.8 (0.7)[ab]	0.010
change	+0.1	+0.0	+0.1	

Abbreviation: PS, protein substitute group; WE, whole egg group; MCV, mean corpuscular volume; FBS, fasting blood sugar; TC, total cholesterol; TG, triglyceride; HDL-C, high-density lipoprotein cholesterol; LDL-C, low-density lipoprotein cholesterol. [a,b] Values in the same row with the same superscript letters are significantly different among the groups at $p<0.05$.
